# Additive Manufacturing of Metal-Infilled Polylactic Acid-Based Sustainable Biocomposites—A Review of Methods, Properties and Applications Abetted with Patent Landscape Analysis

**DOI:** 10.3390/polym17111565

**Published:** 2025-06-04

**Authors:** Sengottaiyan Sivalingam, Venkateswaran Bhuvaneswari, Lakshminarasimhan Rajeshkumar, Devarajan Balaji

**Affiliations:** 1 Department of Mechanical Engineering, J.J. College of Engineering and Technology, Tiruchirapalli 620009, Tamil Nadu, India; sivalaiyaa@gmail.com; 2 AU-Sophisticated Testing and Instrumentation Centre, CoE—Advanced Materials Synthesis and Department of Mechanical Engineering, Alliance School of Applied Engineering, Alliance University, Bengaluru 562106, Karnataka, India; bhuvanashankar82@gmail.com; 3 Department of Mechanical Engineering, KPR Institute of Engineering and Technology, Coimbatore 641407, Tamil Nadu, India; balaji.ntu@gmail.com

**Keywords:** PLA–metal filaments, additive manufacturing, fused deposition modeling (FDM)

## Abstract

Innovations in additive manufacturing (AM) methods represent a significant advancement in manufacturing technology, opening new avenues for creating objects in various shapes and sizes. Fused deposition modeling (FDM) is a specialized AM technique in which computers build layers upon each other to form a complete 3D object. The feasibility of producing metal parts using these methods has been thoroughly analyzed, but the design process has yet to catch up with manufacturing capabilities. Biodegradable aliphatic polyester PLA is derived from lactic acid. To enhance its strength, PLA is combined with metal particles, resulting in versatile property improvements and applications. While the aesthetic and functional qualities of PLA–metal composite filaments are intriguing, they also present difficulties related to extrusion, equipment wear, and maintaining consistent print quality. These challenges could be mitigated, to some extent, with careful tuning and specialized hardware. However, the inferior mechanical properties of bioresorbable PLA filaments highlight the need for the development of infilled PLA filaments to improve strength and other characteristics. This review discusses the 3D printing of PLA infilled with metal particles, various materials used, and their properties as a matter of interest in AM technology. Additionally, the applications of PLA–metal composites, along with their implications, limitations, and prospects, are comprehensively examined in this article. This sets the stage for the development of high-strength, sustainable materials for use in a range of engineering and technology fields.

## 1. Introduction

**Figure 1:**
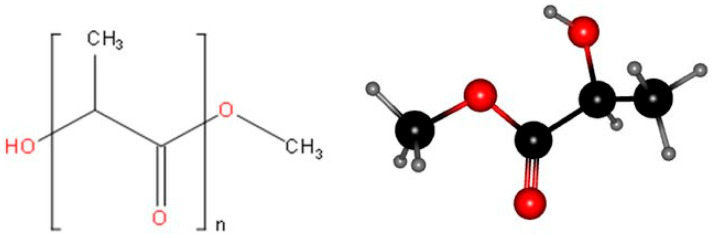
Molecular structure of PLA [9].

Additive manufacturing (AM) techniques normally use three-dimensional (3D) models to create physical objects. Fused deposition modelling (FDM) is an additive manufacturing technique that builds a final object layer-by-layer by melting the thermoplastic filament through a heated nozzle and depositing it over a platform. The built layers then cool to form the final object, which then undergoes post-processing operations. There has been much speculation regarding FDM technique’ explosive growth in use after 2010, and regarding their potential to completely alter the way many industries produce final products [1,2,3,4,5]. Polylactic acid (PLA) is a commonly used source material for FDM-based 3D printing. Nevertheless, the FDM technique has its limitations when it comes to using pure PLA polymers, including with regard to its mechanical weakness and water solubility rate, among others. Figure 1 shows the chemical structure of PLA. Once a product is used, it could be recycled by either melting it down and processing it again or breaking it down into lactic acid, a basic chemical [6,7,8]. As a result, one practical way to improve the qualities of the FDM-produced 3D-printed PLA components is to prepare PLA composites with appropriate additives. The use of FDM in various applications and genuine PLA polymer’s extensive use in 3D printing have been covered in numerous high-quality review articles [1,2,3,4,5,6,7,8].

Polylactic acid (PLA) is unique in additive manufacturing because it comes from renewable sources like sugarcane or cornstarch, is easy to work with, and is good for the environment. This makes it an environmentally friendly alternative to plastics made from oil. PLA, in 3D printing, is better for making complex shapes and personalized products than traditional methods like injection molding because it allows for more design freedom, faster prototyping, and less material waste [10]. Its popularity comes from a few main factors, including its ease of processing, making it a popular choice among both beginners and experts. It melts at a low temperature, does not require much printer calibration, and works with most FDM 3D printers without the need for extra tools. PLA also has a smooth surface and good resolution for small details, which is great for making prototypes or visual models where aesthetics are important. Furthermore, PLA does not give off much of a smell or harmful fumes when it is printed, which makes it a better choice for indoor use. Additionally, PLA is among the most affordable filament options available in the market, contributing to its widespread popularity. It is easy to use and works with many printers, so it could be used by many people [11]. There are clear pros and cons to both additive manufacturing with PLA and more traditional methods of production, such as injection molding. One of the best things about PLA-based 3D printing is that it has a much lower setup cost because it does not require expensive molds or complicated tools, which are needed for injection molding. This makes PLA perfect for low-volume production and rapid prototyping with a focus on speed and low costs. Furthermore, PLA makes it easy to change and customize designs, which gives a lot of freedom and customization options that are not always possible or are expensive in traditional manufacturing. Despite the advantages, the brittleness and low glass transition temperature of PLA prevent the use of 3D-printed PLA composites in high-stress and high-temperature environments.

Additive manufacturing also cuts down on the time it takes to make prototypes, which allows design changes and product development to happen rapidly [12]. Furthermore, PLA printing creates very little waste material, in contrast to the large amounts of waste that are usually created by subtractive or molding-based manufacturing [13]. Overall, PLA is still a useful material for additive manufacturing because it is easy to work with, lasts a long time, and works well, especially for prototypes and small-scale production [14]. The compatibility of PLA with AM technologies allows for a high degree of design flexibility, encouraging innovation and rapid development cycles across various industries [15]. In the evolving landscape of additive manufacturing, material selection is increasingly shaped by a combination of conventional and emerging criteria. While mechanical properties remain a critical consideration, growing emphasis is now placed on factors such as ease of manufacturing, environmental sustainability, and recyclability. Though PLA is characterized as and considered to be a potential candidate in the present scenario, looking to the future, polyethylene terephthalate (PET) is expected to garner more attention, particularly as ongoing research focuses on its potential for efficient chemical recycling and upcycling. These advancements highlight a broader shift toward sustainable materials in advanced manufacturing processes [16].

Biocomposites made by PLA are gaining significant attention due to their durability and sustainability. By combining natural fibers with PLA, these composites offer several advantages, including lightweight structures, cost-effectiveness, and a reduced environmental footprint. Additionally, they exhibit favorable mechanical and thermal properties, making them suitable for a wide range of applications such as food packaging, medical devices, and construction components [17]. Traditional manufacturing techniques could be effectively used to produce PLA-based composites that are not only mechanically strong but also structurally stiff. Life cycle assessments have revealed that PLA consumes approximately only one-tenth of the fossil fuel energy required to produce synthetic polymers. This significant reduction in energy consumption, combined with the material’s biodegradability and performance capabilities, makes PLA a leading choice for the development of environmentally responsible and naturally degradable composites [18].

There is a dearth of review articles detailing the primary procedures for obtaining PLA infilled with metallic particles as composite filaments and their applications in FDM-based 3D printing technologies [19,20]. Aviation, tissue engineering, sensor, battery, robotics, automotive, bioprinting, electrical conductivity, smart textiles, and biomedical industries are just a few more places where newly developed PLA composites have improved the qualities of 3D-printed objects [21]. Addition of metal particles slightly reduces the tensile strength and increases the thermal conductivity of the PLA-based biocomposites. However, reinforcement of continuous metallic fibers mitigates this issue. Meanwhile, addition of more than one metal into the PLA may result in incompatibility and diminishes the properties of the biocomposites drastically [22,23,24,25,26,27,28].

Common AM techniques include inkjet 3D printing, selective laser sintering, 3D printing stereolithography, and FDM. Because of its low production costs, ease of usage, and accessibility, FDM has quickly become the 3D printing method of choice for those on a tight budget. It has also been noted that, for the melting or fusion of metallic particles during layer-by-layer printing, high energy is required, which is possible only achievable with techniques such as SLS, DMD, and SLM, but these methods require more electrical energy, tailored environments and more safety considerations. Due to these constraints, utilization of FDM has been wide and common [29,30,31,32,33,34,35,36,37,38]. This review article comprehensively discusses the extrusion of PLA-based filaments infilled with metal particles using suitable methods. Discussions regarding 3D printing of PLA metal filaments are also made to understand the process parameters used during printing. Applications, limitations, and implications in full-scale implementation of metal-infilled PLA biocomposites are emphasized, while the future scope for this material has been analyzed with the help of patent landscape analysis.

## 2. Filament Extrusion for 3D Printing of Composites

PLA filaments are infilled with metallic particles, such as nickel, iron, cobalt, magnesium, titanium, and so on, through suitable dispersive methods. Commonly, twin-screw extruders have been used to extrude filaments from a hardened steel nozzle for further 3D printing. Addition of metallic particles into PLA as infill improves the mechanical, thermal and biodegradable properties of the biocomposites [39,40]. The following section deals with the extrusion of 3D printing filaments using PLA infilled with various metals.

### 2.1. PLA–Magnesium

**Figure 2:**
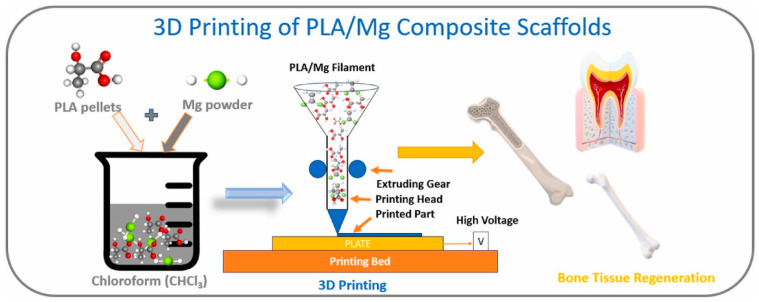
Scaffold preparation [45].

**Figure 3:**
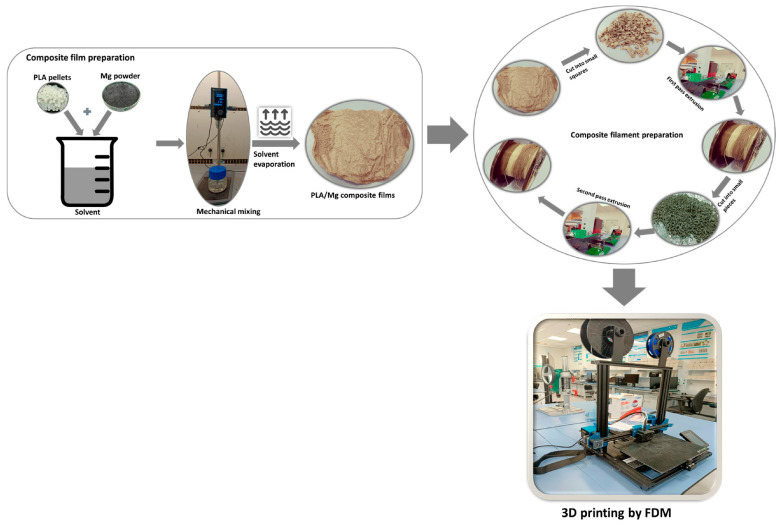
Method of preparing composite filament [46].

Composites based on PLA and magnesium (Mg) show promising use in bone regeneration, and also in tissue engineering. PLA is a polymer which is easily convertible to various forms including fibers, scaffolds, and films. It is both biodegradable and biocompatible. But it cannot be used for tissue engineering because of its poor natural biodegradation properties. In contrast, bone tissue engineering works wonderfully with magnesium because of the biocompatibility of the metal and its excellent osteo-conductivity and biodegradability [41]. Using the two-stage extrusion method, researchers have generated PLA filaments reinforced with varying proportions of Mg microparticles. The goal is to study the effects of treating on the thermal degradation of these filaments. Researchers also conduct in vitro degradation experiments, with the Mg microparticles completely released upon 84 days within phosphate-buffer saline media. The primary objective is to obtain a usable filament for future 3D printing; it makes sense that the product will be more scalable, and the processing is simpler. Here, authors achieve micro-composites by means of the double-extrusion technique; the materials are preserved throughout, and the microparticles are evenly distributed throughout the PLA matrix [42]; and the microparticles are not subjected to any physical or chemical alterations [43]. This demonstrates that it is feasible to obtain PLA bio composites filled with Mg as a filament feedstock for AM based on material extrusion. Using these materials in medical applications allows for the full customization and design freedom that AM technology offers. Samples for testing and ACL (anterior cruciate ligament) screws have been produced using filaments made from two PLA + Mg + vitamin E (α-tocopherol) combinations using a cheaper 3D printer [44]. Figure 2 shows the scaffold preparation with PLA/Mg [31]. Figure 3 shows the method of preparing composite filament with PLA/Mg [31].

### 2.2. PLA Zinc

Biocomposites of polylactic acid and zinc oxide (ZnO) for use in tissue engineering of bone scaffolds printed using 3D printing technology are developed. For regulating the inhibitory impact of ZnO upon the decomposition caused by PLA throughout the molten state processing, the surface of ZnO was functionalized using maleic anhydride (ZnOMA) using radio frequency plasma treatment. The scaffolds were 3D printed from biocomposites by heating PLA with ZnOMA powders in an internal mixer.

**Figure 4:**
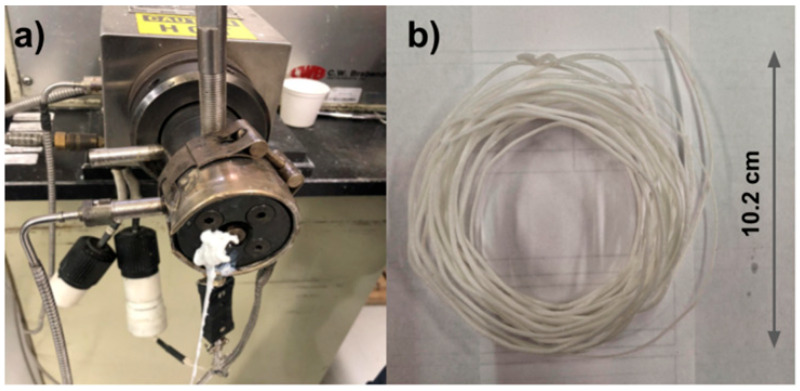
Filament Extrusion Process: (**a**) extruder clogging at higher wt. % of ZCB; (**b**) resultant PLAC10 filament with 10% ZCB [49].

Using PLA composites doped with zinc oxide (ZnO) and silicon carbide (SiC), the creation of triboelectric generators, a type of gadget that transforms mechanical energy from their surroundings into electrical energy was investigated. FDM printing technology makes heavy use of PLA, a biodegradable thermoplastic polyester extracted from renewable resources, including sugarcane or corn starch, due to its low impact on the environment and good mechanical properties [47]. Researchers are looking into the addition of nanofillers like ZnO and SiC to PLA, which could improve its natural properties and boost the electricity generated by nanogenerators that use PLA. ZnO was added to the composite because it has many useful features like UV protection, germ-fighting properties, and better heat conduction, while SiC was chosen as a dopant because it is very hard and strengthens the material. This study thoroughly evaluated the electrical and mechanical characteristics of PLA materials doped with SiC and ZnO to better understand how these materials could improve the performance of triboelectric generators for use in environmental energy harvesting [48]. Figure 4a,b shows the extruder clogging at higher wt. % of ZCB and resultant PLAC10 filament with 10% ZCB, respectively.

### 2.3. PLA Copper

The effects of using copper additives on the breaking and micro-mechanisms in 3D-printed PLA samples subjected to combined mode one/two loading were examined. Pure PLA and PLA/Cu samples, which were pre-cracked and made with the FDM techniques, are put through a series of tests to compare their fracture behavior. There were three distinct orientations for the layers used to fabricate the SCB specimens. Copper additives raised the load-carrying capacities (LCCs) of pre-cracked SCB samples with flat, on-edge, and erect layer arrangements by 2.9%, 16%, and 27.7%, respectively, according to the compared investigational results. Lastly, SEM is used to shed light on how the micro-mechanisms of breakage are affected by copper additives [50]. Samples were printed one at a time and placed in the center of the construction frame [51].

### 2.4. PLA Bronze

**Figure 5:**
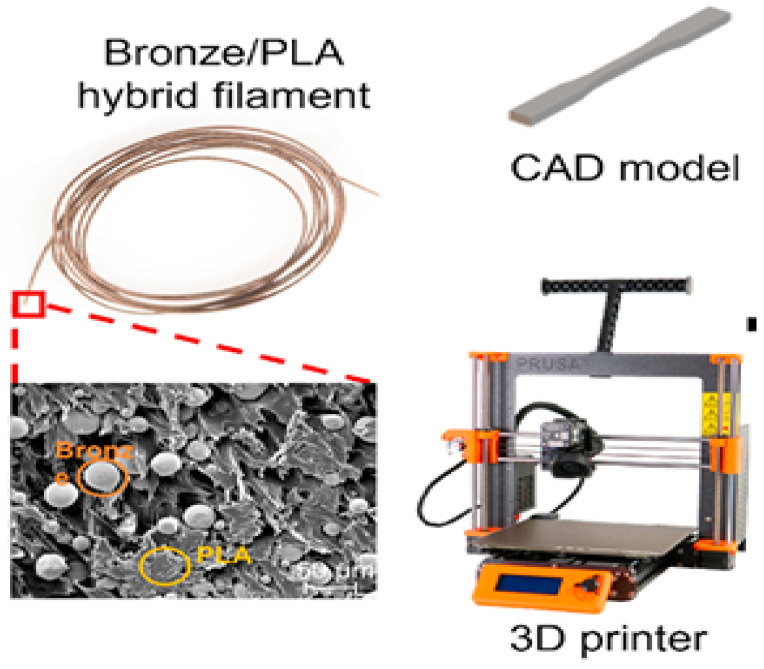
Filament extrusion of PLA Brass [54].

During the hot extrusion process, 14% bronze composite filament was extruded by using PLA through the FDM technique. The samples measure 50 × 50 × 10 mm, with a filled density of 100% and an orientation of 45°. By printing two separate specimens, one with and one without holes, the printing status and the printed specimen’s response to micro drilling were compared. The commercial drill is used with a predetermined cutting width, speed, and feed. Scanning electron microscopy is used to verify the size of the drill hole. An investigation and report are conducted on tool wear and the condition of the drilled hole. Based on observation, it is evident that, to avoid secondary machining operations with holes smaller than one millimeter, this constitutes an inevitable requirement [52]. The machining, cutting, and feed speed affect both the surface delamination region and the diameter of the hole circumference [53]. Figure 5 depicts the PLA/Brass Filament extrusion [40].

### 2.5. PLA Brass

The mechanical characteristics of an FDM 3D-printed composite material composed of PLA and brass were examined. One component of this composite material is brass, which is known for its outstanding mechanical properties, and the other is PLA. This study evaluated the chemical and physical properties of the PLA/Brass (PLA/Br) composite using different analysis methods, including FTIR, SEM, and Energy-Dispersive X-ray Spectroscopy (EDX) [55,56].

### 2.6. PLA Titanium

Despite the development of titanium filament for use in FDM techniques, little is known about the mechanical characteristics of objects printed with this material. Three-dimensional printing filaments consisting of titanium are possible. The FDM technique was used to print the composites containing PLA incorporated with titanium. The primary goal of this research is to determine the 3D printing strength of this new filament. The study made use of four different 3D printing parameters, each of which included three layers. The approach of Taguchi L9 (3^4) is utilized in the experimental design [57]. It was possible to create porous, three-dimensional (3D) PLATi6Al4V (Ti64) composites with interconnected channels using a material extrusion approach.

### 2.7. PLA Iron

**Figure 6:**
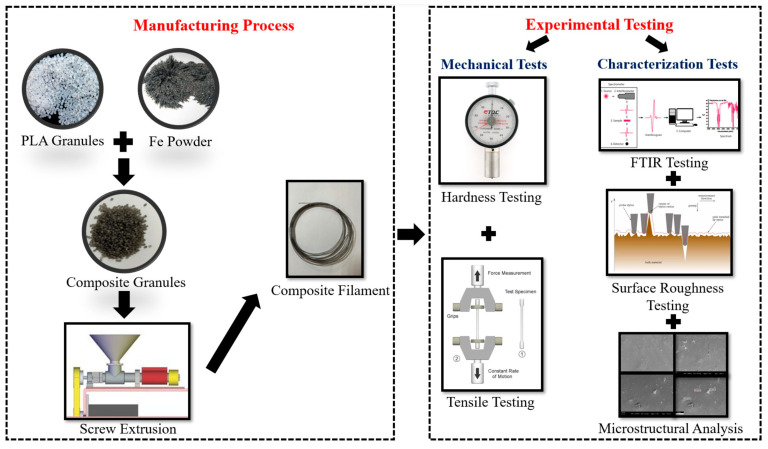
Manufacturing process and experimental testing method [59].

Metal particle reinforcement greatly affects the printability and mechanical qualities of composites when using the FDM method. PLA/iron composite filament finds numerous applications, including in magnetic and biological devices. FDM is a widely used 3D printing method for producing thermoplastic components. Although this approach is useful in creating complex structures with excellent mechanical and wave-absorbing characteristics, its application in developing and manufacturing radar wave-absorbing structures is rare. The validity of the method was demonstrated by loading carbonyl iron particles into FDM-fabricated thermoplastic composites [58]. PLA Iron filaments were extruded, and the manufacturing methods are shown in Figure 6.

### 2.8. PLA Silver

**Figure 7:**
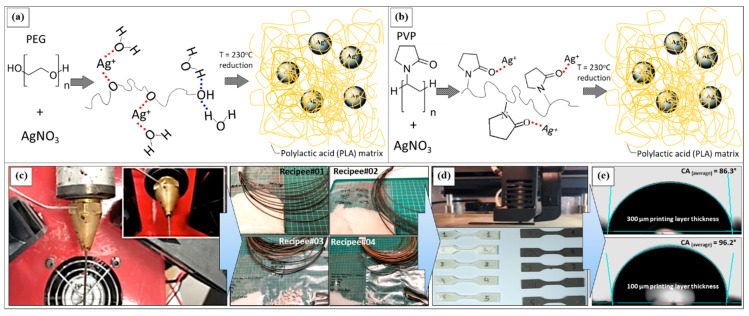
PLA with silver composite (**a**,**b**) chemical structure and reaction to form PLA/Ag biocomposites, (**c**) Filament extrusion, (**d**) Tensile test specimen, (**e**) Contact angle measurement [60].

Researchers have explained a method for mixing PLA and silver nanoparticles (AgNPs) directly in the melt, which could be used widely in industry to create nano-composite filaments. At the start of making PLA/AgNP nano-composite filaments, silver ions were converted from silver nitrate (AgNO_3_) in the melted polymer into silver nanoparticles. Researchers achieved this transformation using either polyethylene glycol (PEG) or polyvinyl pyrrolidone (PVP), both of which are reducing macromolecular blend compounds. Researchers supplemented the PLA matrix with varying quantities of PEG and PVP. Using PLA/AgNP filaments, 3D-printed antimicrobial components were made using FDM technique. Researchers analyzed the influence of 3D-printed PLA/AgNP parts for the survival of *Staphylococcus aureus* (*S. aureus*) and *Escherichia coli* (*E. coli*) bacteria after 30, 60, and 120 min of contact to identify the key antimicrobial features. Figure 7 shows that the AM components tested herein imitate nature’s mechanisms through bacteria. Due to their manufacturing process, their ability to interact with surfaces to prevent adhesion, and their inherent antimicrobial properties, these parts could pave the way for the upcoming development of low-cost healthcare, on-demand PPE, and nosocomial devices [60].

Many efforts have sought to enhance the stability of 3D printing materials or give them new, specific features. Nanomaterials offer wonderful functionality, which is why 3D printing with them is becoming increasingly popular. Additive manufacturing process for making antibacterial filament from PLA and silver nanoparticles was dealt in some papers [61]. After making the polymer dope of silver salts in PLA using both solution–phase and solvent-less methods, the next step was to extrude the mixture into filaments. Silver salt decomposed in situ during the filament’s heat extrusion, creating silver nanoparticles. An XRD and TEM analysis verified the nanoparticles’ production and distribution throughout the polymer framework [62]. This study proposed a method using the liquid form to prevent problems caused by how nanoparticles spread during melt mixing. Researchers prefabricated specimens using the filament and subjected them to mechanical and microbiological testing [63]. Things that could be printed out showed promise as antimicrobials when tested against common bacteria, including *Pseudomonas aeruginosa*, *E. coli*, and *S. aureus.* The study found that adding silver nanoparticles to PLA (in amounts from 0.01 to 5 wt. %) does not change its overall properties, but makes the material fight against germs. Production of personalized antimicrobial items on demand could potentially utilize the filament [64].

### 2.9. PLA Cobalt

This study set out to create a biodegradable substrate that could work in conjunction with a colorimetric humidity indicator to detect moisture in food packaging. The researchers made PLA stronger by melting and mixing it with safe poly (ethylene oxide) (PEO) at 180 °C. They created three-dimensional (3D) PLA/PEO substrates using solvent-cast 3D printing. Additionally, a cobalt chloride (CoCl_2_) solution was printed onto the substrate using an inkjet printer. This solution could be used later as a colorimetric indication for humidity detection [65].

### 2.10. PLA Nickel

The matrix material was PLA, and two reinforcing wires with a diameter of 75 µm each were utilized: one was nickel-chromium wire, and the other had coatings of copper magnet wire. Experiments showed that using a 75 µm diameter wire with a 1 mm nozzle, a 0.6 mm layer height, a speed of 7 mm/s, and a nozzle temperature of 175 °C usually produced consistent prints. To make unidirectional specimens with the specified dimensions, the printer was operated using a modified G code. This method removed wires that intersected in the same layer. The printing process for these samples starts at one corner of each layer and continues to another corner before moving on to the next layer [66].

## 3. Additive Manufacturing of Pla–Metal Biodegradable Composites

**Figure 8:**
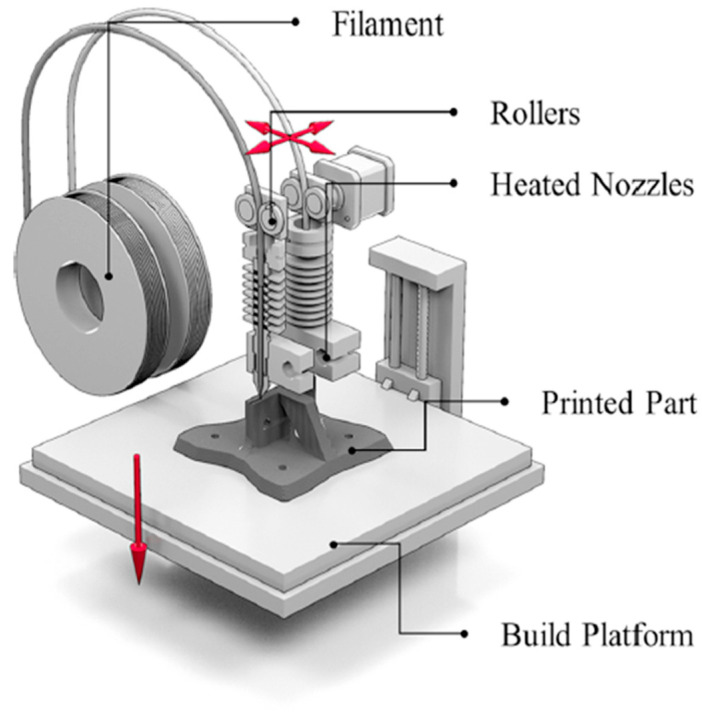
Schematic of FDM technique [67].

For the manufacture of the PLA–Metal biodegradable composites, the FDM technique was the most commonly used method. The FDM technique works on the principle of building a solid object through layer-by-layer deposition of the melt filament fed via a nozzle on a platform heated to a certain temperature to enhance the solidification rate of the 3D printed part. This method could handle PLA-based filaments with reinforcements in fibrous (long and short), and particulate forms. Figure 8 depicts the schematic of the FDM technique.

Researchers prepared PLA-Mg filaments for various biomedical applications and manufactured the films using the AM technique. After preparing them as films, the extruder chopped the PLA/Mg composites in varying concentrations into even-sized squares. In this case, the most cutting-edge filament extruder was a Filabot EX2 system. The system employed melt-integrating extrusion, operating at an average temperature of 175 °C, to push the filaments through a 0.2 mm nozzle. The filament that had been extruded was subsequently ground into tiny, even pieces and put back into the extruder to confirm that the Mg particles had been consistently distributed. Subsequently, the filaments were utilized with different amounts of magnesium for additional research and 3D printing [46]. Few studies have used PLA-Zn-based composites and used AM techniques to manufacture composites. The process maintained a 100% infill density and a concentric infill pattern. The first layer thickness is 0.3 mm, whereas the subsequent layer thickness is maintained as 0.2 mm. The first layer is printed with a 30 mm/s speed, whereas the layer print speed is maintained at 80 mm/s. The nozzle temperature is set at 230 °C, while the bed temperature is maintained at 60 °C. We set the build orientation along the x–y plane in a flat configuration. The nozzle diameter was set to 0.4 mm for seamless printing [68].

It was stated in various studies that the AM techniques could process complicated parts with accurate dimensions at the lowest feasible cost; FDM has seen extensive use in additive manufacturing. Materials used in FDM, like PLA, have low melting points because of the technology’s limited working temperature range. The problem with FDM-printed thermoplastics is that they do not have the mechanical strength or properties needed to print functional parts, like thermal and electrical conductivity. These problems have led to the development of a novel composite material for the FDM technique. Investigation performed on the flexural properties of polymer-matrix composites (PMCs) with varying amounts of copper reinforcement (25 wt. % and 80 wt. %) using various infill designs. The study focuses on examining the differences in input velocity variables between PLA and PLA/Cu composite filaments. The study introduces a numerical model of a nozzle and uses finite element analysis to examine the behavior of the melted filament fluid. The study thoroughly examined the flow characteristics, including pressure, melting temperature range, and velocity. Next, tests were conducted to determine the optimal printing velocities for the two filaments [69]. Changing the filament feed velocity causes a corresponding change in the flow parameters, according to the results. Lastly, the ideal input velocity for PLA is from 2 to 3.5 mm/s, while for PLA/Cu, it is less than 5 mm/s, as determined by the assessment of printed specimens’ dimensional accuracy [70].

A readily accessible PLA/Cu filament was 3D printed employing the FDM technique. The tensile properties of this filament were the primary focus of this investigation. Printing orientation and fill pattern, along with infill density, were among the many parameters for printing tweaked to achieve this goal [71]. Usually, 3D-printed samples are tested for strength to see how the printing settings affect the strength of the printed parts. According to the findings, a distinct fracture mechanism occurs when printing horizontally rather than vertically, resulting in stronger components for the material under investigation. More infill density and an ordered fill pattern both contribute to increased mechanical strength [72]. Four key parameters related to FDM were primarily examined: layer thickness, infill density, nozzle temperature, and manufacturing speed. Generated for the FDM method was a 14 wt. % nano copper reinforcement with PLA. The prepared filament was constructed according to ASTM standards and subjected to various parametric conditions to learn about the mechanical characteristics exhibited by the composite filament and the impact of FDM parameters [73]. Experimenters investigated and reported the influence of layer height, bed temperature, and nozzle temperature on compression and flexural behavior of the 3D printed samples. A rise in both compressive and flexural strengths has resulted from the heated bed and nozzle [74]. In various experimental studies, PLA–bronze filaments were used for the manufacturing of 3D printed composites. The test pieces were produced using the commercial 3D printer WANHAO Duplicator 6 with a nozzle diameter of 0.4 mm in the additive manufacturing laboratory at Szent Istvan University, Hungary. The specimens were printed with a 45°/135° raster angle. The printing temperature and platform temperature were maintained at 195 °C and 60 °C, respectively. Before testing, the specimens were conditioned in a climate room with a temperature of 25 °C and relative humidity of 50% [75].

For obtaining intact 3D printed composites using PLA–brass filaments, examiners meticulously manipulated printing parameters, such as layer height and printing speed, on a Raise3D N2 Plus FDM 3D printer to produce test specimens with varied compositions. Layer height values span 0.25 mm, 0.30 mm, and 0.35 mm, and the printing process speeds range from 20 mm/s to 40 mm/s. The compositional variables range from 15 to 80 wt. %. Find out how the printing parameters affect different mechanical characteristics with the help of this research. For instance, it was noted that the optimal printing parameters for achieving the highest elastic modulus in tensile testing were a layer height of 0.25 mm and a manufacturing speed of 30 mm/s [76]. Similarly, the study clarifies how to modify additive manufacturing parameters to enhance the properties of PLA/brass composite materials for diverse applications. In addition to expanding the research understanding of PLA/brass composites, an opportunity to develop novel materials was also explored [77]. Research works focused on the manufacturing of 3D-printed composites using PLA–titanium filaments with varying process parameters such as nozzle temperature, layer height, infill density, and bed temperature. The analysis of variance (ANOVA) showed that the following factors significantly affected the tensile test results: nozzle temperature (19.9%), layer height (1.9%), bed temperature (39.5%), and infill density (28.5%), listed from most to least important. A combination of a 224 °C nozzle temperature, 0.2 mm layer height, 41 °C bed temperature, and 95% infill density produced the maximum tensile stress of 15.4 MPa [57].

For printing PLA/aluminum, the following three control input parameters were provided: the number of shells, infill density, and layer thickness. There were three possible values for the following parameters: layer thickness (0.1, 0.2, and 0.3 mm), number of shells (2, 3, and 4), and infill density (20%, 40%, and 60%) [78]. To produce the PLA/Iron (Fe) and PLA/stainless steel (SS) composites, the printing parameters include an infill density of 100%, printing rates ranging from 30 to 90 mm/s, and temperatures between 260 and 290 °C [79]. In order to manufacture 3D-printed PLA/silver composites, investigators constructed and used a small extruder with a short heating duration of 3 cm to reduce thermal deterioration during TS-SOC filament production [80]. Additionally, they generate dense conductive percolation networks by uniformly forming silver nanoparticles (SNPs) with a diameter of 70–90 nm through surface dechelating and chemical reduction. A few studies have been carried out on PLA–cobalt biocomposites. Recommended printing parameters according to the materials data sheets are employed, which include a printing temperature of 210 °C for both materials, a heated printing bed at 40 °C, a printing speed of 20 mm/s for the initial layers and 40 mm/s for the intermediate and upper layers of the constructs, and a layer height of 200 μm. An infill of 100% is applied to work with bulk materials and promote adhesion within and across layers [81]. PLA/nickel filaments were used for the manufacturing of 3D printed composites by various researchers. Experiments showed that using a 75 µm thick wire with a 1 mm nozzle, a layering height of 0.6 mm, a speed of 7 mm/s, and a nozzle temperature of 175 °C usually led to consistent prints [82]. Table 1 enlists various 3D printing process parameters for the manufacturing of PLA–metal biocomposites.

## 4. Properties of 3D Printed Pla–Metal Biocomposites

### 4.1. Mechanical Properties

**Figure 9:**
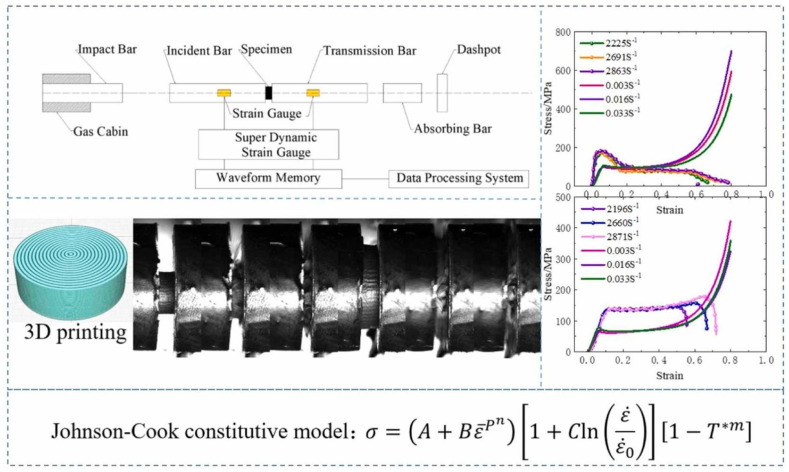
PLA with Copper composite analysis for mechanical behavior [95].

One way to make AM PLA stronger, since it was not strong enough for many real-world applications, is to add copper powder to make a new material called PLA-Cu. A universal testing machine and Hopkinson pressure bar equipment were used to examine how PLA and PLA-Cu respond to different types of stress [93]. This experimental study developed a constitutive model based on the mechanical behavior of each material. Impact embrittlement influences PLA-Cu, meaning it acts in a straightforward, predictable way when under fast-moving conditions [94]. Both the composite and the pure PLA react differently to changes in speed from static to dynamic condition, but adding copper powder significantly increased the strength needed to make them deform due to the ductility and load-bearing capacity of the copper. To better predict how these materials behave under different loads, the Johnson–Cook constitutive equation was adjusted by including the Cowper–Symonds strain rate term. The results provided valuable information for numerical models for PLA with copper in Figure 9 [95].

The effect of FDM settings on the compressive strength of composites with PLA and copper filler. Researchers examined the impact of changing process parameters on flexural, tensile, and impact strengths [96]. By manipulating the process parameters, mathematical models were created to forecast the composite strength. As the printing speed and thickness of the layers increased, the composites’ strength decreased. Conversely, the composites’ strength was enhanced by raising the nozzle temperature along with infill density. To gain insight into the mechanisms of fracture, characterization analysis was applied to the composite samples. The specimens that are affected by the method parameters that affect layer bonding and porosity exhibit both brittle and tensile modes of failure [97]. To find out the strength of two important materials, three different layer arrangements of PLA and PLA/Cu were used to create and test dog bone samples. The test results show that adding copper to 3D-printed PLA samples increases their maximum tensile strength by about 16% when flat, 14% when on the edge, and 10.4% when standing upright. The LCCs of samples with pre-existing cracks could be estimated using the Equivalent Material Concept (EMC) along with the Average Strain Energy Density (ASED) method, as well as through actual testing. By using the EMC-ASED criterion, the measured LCCs of FDM-PLA and FDM-PLA/Cu samples could be accurately estimated [50].

Understanding the fundamental mechanics of FDM-printed parts is essential for engineers. Because of its low cost and precise dimensional processing capabilities, FDM has seen extensive use in AM for processing complicated parts. The filament-shaped thermoplastic polymers are the main components. The FDM method, applied to PLA and PLA/Cu, aims to develop testing samples with different infill percentages (25, 50, and 75%) and designs [98]. After that, tensile testing is employed to evaluate the mechanical characteristics of copper. The results indicate that the best tensile strength performance was achieved with the infill design using a triangle PLA/Cu sample and a concentric PLA specimen with a 75% infill percentage. In contrast, the grid copper design exhibited the least desirable characteristics of all the patterns [99].

**Figure 10:**
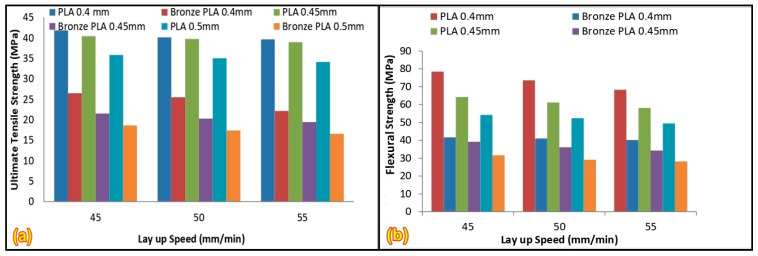
Variation of strength of PLA/Bronze specimen (**a**) Tensile strength, (**b**) Flexural strength [102].

Producing PLA and bronze-filled PLA (PLA/BZ), the latter of which has lower flexural and tensile moduli, is made possible by 3D printing technology. The functionality of 3D-printed components rely heavily on the material’s mechanical properties, which are crucial for their use in engineering [100]. The specimens were prepared using an AM employing commercially available PLA and PLA/BZ. Mechanical characteristics, including tensile and flexural strengths, were examined in the samples. Three-dimensional printing was used to fabricate specimens with varying layer heights and build speeds. Research findings were used to compare PLA and PLA/BZ, and the results revealed that PLA had better tensile and flexural properties [101]. Researchers created samples of PLA and bronze-filled specimens using various layup process parameters to assess their mechanical properties. The dog bone piece for the tensile test is depicted in Figure 10.

The printing parameters affect the printed specimens’ mechanical behavior. The investigation examines aspects such as bending, compression, tensile, and impact resistance. Even now, 3D printing filament—particularly filaments containing a combination of metals—can be quite costly. The author used a DIY extruder to make PLA and brass filament. The accuracy and durability of the filament define the success criteria. The printed filament’s accuracy was checked with a 0.01 mm micrometer, and its strength was determined by administering the pull-out test. The following three parameters are utilized, with each two levels: barrel temperature, material composition, and roller speed [55].

This research aims to look at how well a PLA composite with iron particles could be printed, how strong it is, and compare it with a PLA composite that has stainless steel particles. The researchers used different printing settings, including filling the material completely, printing speeds from 30 to 90 mm/s, and temperatures between 260 °C and 290 °C, to create the PLA/iron (Fe) and PLA/stainless steel (SS) composites. Printed PLA/Fe composites had 1.5 times the maximum stress and 1.2 times the elongation of printed PLA/SS composites. Furthermore, when printing at 280 °C and 60 mm/s, the PLA/Fe composite specimens achieved a maximum tension of 40.20 MPa. Researchers observed the printed structure and the uniform distribution of Fe and SS particles throughout the PLA composite using an optical microscope [103]. Achieving the required outcome requires studying the mechanical characteristics and influence of the printing settings on printed pieces of different materials. Aiming to compare PLA and PLA/aluminum composite tensile strengths, Taguchi’s design of experiment (DOE) technique was used to manipulate various printing settings. The results demonstrate that PLA outperforms the PLA/aluminum composite in terms of tensile performance. The ANOVA study primarily affects the tensile strengths of both materials based on the number of shells [104].

Flexural test findings showed that the two parameters have a significant impact on flexural characteristics. The flexural test yielded the initial flexural modulus and flexural strength values. Specimens with a 25 wt. % Cu composition and a concentric infill pattern have a flexural strength of around 26 MPa [105]. The composite specimen was subjected to tensile, flexural, and impact strength tests. Analysis of variance was used to test the accuracy of the mathematical models that have been created using response surface methodology [106]. Raising the nozzle temperature and infill density enhances the composite’s strengths. As printing rate and layer thickness increased, the composite specimen’s strengths diminished. Although other parameters contribute to impact strength, infill density (58.12%) is the most important factor determining tensile strength (30.82%), flexural strength (44.02%), and other parameters, in that order. The examiners recorded the maximum values for flexural strength (19.3 N/mm), impact strength (0.26 kJ/m), and tensile strength (36.08 N/mm). Given the current limitations of the FDM process when it comes to brass alloy properties, this study aims to fill that gap by analyzing the bend and compression characteristics of FDM-printed parts with different infill patterns and 15% and 70% wt. of brass-reinforced PLA from an examiner’s perspective. The relevant ASTM standards guided both the sample processing and analysis.

Compared with casting or sintering materials, the mechanical nature of 3D-printed objects will be inferior due to the weak connection between successive layers. The orientation among the printed layers might therefore significantly impact the products’ behavior. The authors of another study mechanically evaluated a 3D-printed composite that uses PLA with bronze filler as an alternative to sintered bronze components under compression loads [107]. The stress–strain curves in ambient temperature and the transitional glass zone were determined by subjecting cylindrical specimens grown having the base horizontally or vertically to compression loads. This study filled a gap in the literature by investigating the potential of bronze-filled PLA as a material for gaskets and other compression-sensitive items [108]. Additionally, bend and compression characteristics were best captured by the grid pattern, while the concentric pattern was determined to be the optimal printing pattern [109]. Although the addition of Ti64 raised PLA’s glass transition temperature, it had a slight negative impact on the filament’s thermal stability, melting point, and crystallization point. Before adding from 3 to 6 wt. % of Ti64, the mechanical properties of PLA were already good. However, PLA-3Ti64’s maximum compressive strength and compressive modulus went up by 50 MPa and 2 GPa, respectively. The flowability tests also confirmed that all composite filaments were print-ready.

**Figure 11:**
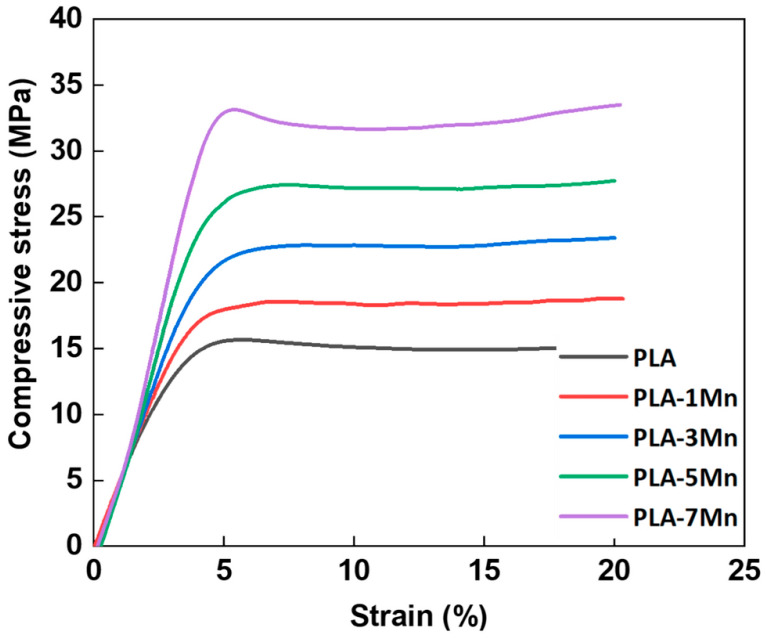
Compressive stress vs. strain for PLA-Mn biocomposites [113].

PLA-ZnOMA additionally obtained elastic modulus values near 1 GPa, making them suitable for use in bone applications [110]. When tested for cell activities, PLA-ZnOMA scaffolds encouraged mesenchymal adult stem cells to change into osteoblasts, and the survival rate of these cells was 160% higher than that of the untreated samples. The untreated samples did not have the right flexibility for bone use, but tests showed that ZnOAM biocomposites did have it. Cell viability was better in PLA/ZnOAM scaffolds compared to PLA/ZnO composites with treated filler surfaces, even though the untreated samples showed more bioactivity [111]. Finally, the tensile test results show that the octa-spiral pattern is the weakest and the concentric pattern is the strongest for both compositions, with the highest values for elastic modulus and ultimate tensile strength as well as yield strength. A maximum of 0.3 GPa, 8 MPa, and 5 MPa were achieved for the ultimate tensile strength, yield strength, and elastic modulus, respectively [112]. Figure 11 shows the compressive stress vs. strain graph for PLA-Mn composites. It was observed from the test results that 7% Mn particles enhanced the mechanical properties of the PLA–biocomposites.

The impact of two compositions and five infill patterns on bend and compression characteristics was evaluated using RSM [114]. Next, the intended bending and compression characteristics are predicted using a prediction model. The results demonstrate that the specimens with a 15 wt. % brass composition exhibit superior characteristics when contrasted with those with a 70 wt. % brass composition. This is because the interlayer bonding energy with the PLA was reduced because of an increase in the weight% of brass composition. Researchers used the FFF method to study how printing speed (PS) and nozzle temperature (NT), along with the usual porosity (POR), affect the impact strength and specific impact strength of PLA/iron composites. RSM improved the experimental design. RSM optimized the experimental design. Based on the statistics, POR significantly affected the impact resistance more than NT. When researchers reduced the POR, the impact resistance of the samples improved. In this multi-objective optimization, examiners identified the optimal conditions for multiple objectives, which included the least NT (190 °C), the lowermost POR (30%), and the PS of 50 mm/s. Researchers also created mathematical models using RSM to understand how changes in NT, POR, and PS affect the desired impact qualities [115]. Both the composite and pure PLA react differently to changes in speed, but adding copper powder significantly increases the strength of the pure PLA [95]. A maximum of 0.3 GPa, 8 MPa, and 5 MPa were achieved for the ultimate tensile strength, yield strength, and elastic modulus, respectively [112].

The Shore polymer-specific hardness test was carried out in a few investigations. Examiners found that the printing parameters impacted the hardness of the printed specimens. Examiners achieved a maximum hardness level of 56.3 Shore D by adjusting the print parameters to 240 °C for the nozzle, 0.3 mm for the layer height, and 30 mm/s for the speed. Dentures constructed from PLA–titanium showcased the practical use [116]. Finally, the tensile test results show that the octa-spiral pattern is the weakest, and the concentric pattern is the strongest for both compositions, with the highest values for elastic modulus and ultimate tensile strength as well as yield strength for PLA/ZnO composites [112]. This investigation provides a relationship between these findings and the ductility of the material resulting from fused filament production. This study looks at how the printing angle (a single processing parameter) changes the mechanical behavior and microstructure of a PLA–iron (PLI) composite that was made to be magnetically active [117]. The results show that the angle at which something is printed greatly affects the strength and direction of the AM PLI composites, and using a 45°/45° layer arrangement gives the best strength [118]. The results show that PLI composites made with 3D printing offer a cost-effective method to create bio-based materials that could change with light and have complex magnetic movement features [117].

### 4.2. Thermal Properties

Researchers create PLA filaments with different amounts of Mg microparticles using a two-step extrusion process. The goal is to study the effects of treating the thermal degradation of these filaments. Researchers also conduct in vitro degradation experiments, with the Mg microparticles completely released after 84 days in phosphate-buffered saline media [119]. The primary objective is to obtain a usable filament for future 3D printing, so it makes sense that the product will be more scalable if the processing is simpler. Here, authors achieve microcomposites by means of the double-extrusion technique; the materials are preserved throughout, and the microparticles are evenly distributed throughout the PLA matrix. The microparticles are not subjected to any physical or chemical alterations [43]. Although the addition of Ti64 raised PLA’s glass transition temperature, it had a slight negative impact on the filament’s thermal stability, melting point, and crystallization point. By reducing the number of conductive fillers, conductive 3D printing could make it easier for the electronics manufacturing business to provide easily customizable parts [120]. In this situation, examiners create a silver–organic complex (SOC) to make a 3D-printed filament material that contains less than 3 wt. % of pre-conductive additives [121].

**Figure 12:**
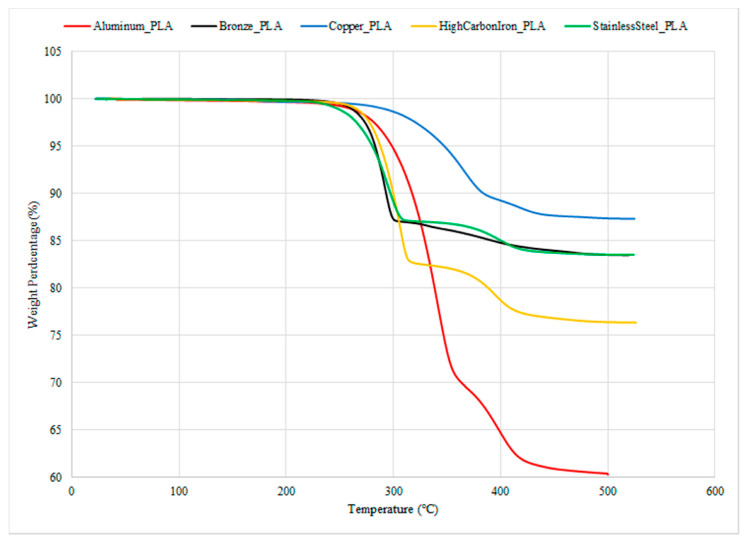
Thermogravimetric analysis of PLA/metal biocomposites [123].

Even when exposed to high heat during extrusion and 3D printing, the cage effect reduces thermal damage to 0.91 wt. % in a thermally stable (TS)-SOC that has chelating agents. In summary, the electrical properties might reach 55.7 S/cm, and the problems of short circuits from nozzle clogs in regular conductive filament could also be fixed. Researchers discovered that adding more PEO to PLA improved its thermal stability and flexibility while increasing its hydrophilicity. Total color difference and picture analysis verified the material’s sensitivity and color variations [65]. Simulations indicate that the setup of a nickel-coated antenna and a silver-coated diplexer has a return loss greater than 10 dB and a high gain efficiency of 12 to 17 dBi in the RX band of 10.7 to 12.8 GHz and the TX band of 17.42 to 18.81 GHz. The measurement findings of the feed system verify these predictions. Additionally, the rejection stage between the TX and RX ports exceeds 60 dB. This low-cost production method for making prototypes or testing ideas for Ku-band systems is shown to be effective because it has a complicated design with many shapes inside, unlike the more expensive CNC-based metal manufacturing methods [122]. Figure 12 denotes the thermal stability curves of different PLA–metal 3D-printed biocomposites.

### 4.3. Characterization

**Figure 13:**
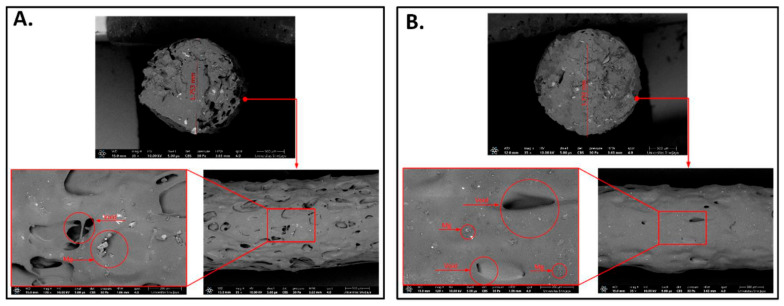
SEM analysis of optimized filaments (**A**) 10% Mg, 7.5% PEG, (**B**) 7.5% Mg, 7.5% PEG [125].

SEM, vibrating sample magnetometers, broad-band dielectric spectrometers, and UTM are some of the tools that researchers use to look at the composites’ mechanical, electrical, magnetic, and morphological properties. The composites show a typical soft ferromagnetic hysteresis loop, which includes a coercive force, hysteresis losses, and very little remanence [124]. The saturation magnetization per mass of composite iron powder is not directly proportional to filler concentration. Composites made using these technologies have far higher total elongations and marginally higher tensile strengths compared to those made using more conventional methods [58]. Figure 13 shows the SEM analysis of optimized PLA Mg filaments.

**Figure 14:**
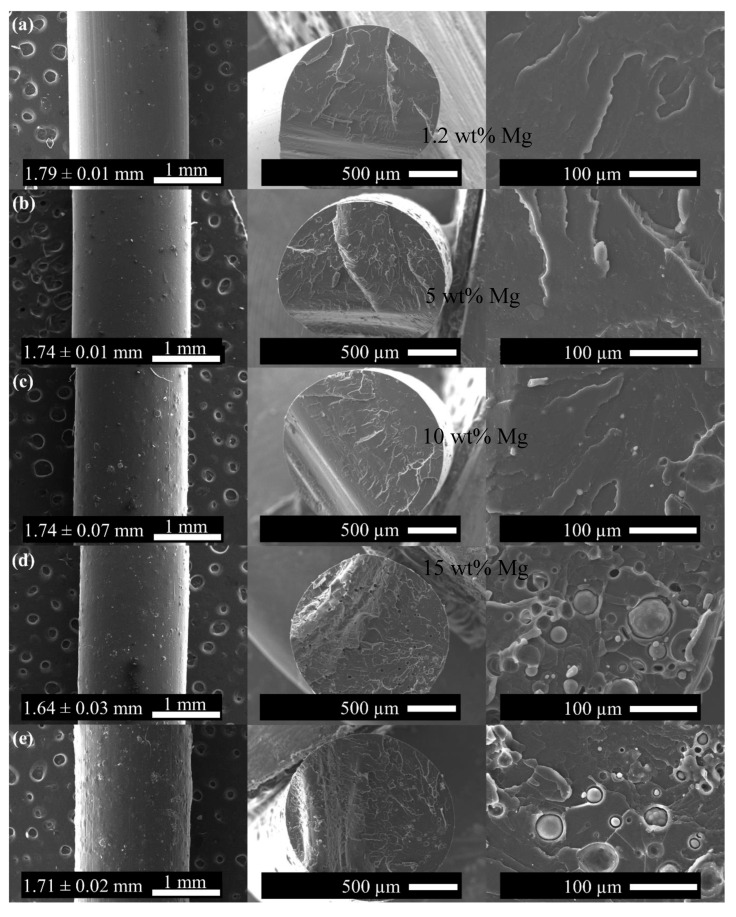
SEM images with different weight fractions of magnesium (**a**) pure PLA, (**b**) 1.2% Mg, (**c**) 5% Mg, (**d**) 10% Mg, (**e**) 15% Mg [43].

X-ray photoelectron spectroscopy (XPS) and Fourier transform infrared spectroscopy (FTIR) have also been employed to study the scaffolds’ deterioration, and thermogravimetric analysis (TGA) and differential scanning calorimetry (DSC) have been used to compare the composites’ thermal characteristics. Researchers revealed that magnesium has a major impact on cell adhesion, drastically changes PLA’s thermal properties, and speeds up its degradation [45]. Figure 14 depicts the SEM images with different weight fraction of magnesium.

**Figure 15:**
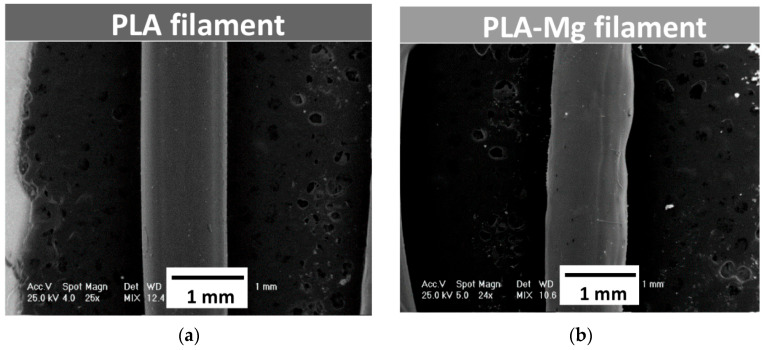
SEM images of PLA and PLA/Mg filaments [44]. SEM images of (**a**) PLA and (**b**) PLA/Mg filaments.

Scanning electron microscopy (SEM), Fourier transform infrared spectroscopy (FTIR), and differential scanning calorimetry (DSC) were used to characterize the filaments and implant screws. A good integration was noted, likely because vitamin E was used as a precursor; however, the filament manufacturing method was unable to ensure an even scattering of Mg particles throughout the PLA matrix [126]. Additionally, the results show that composite biomaterials could maintain the strength of implant screws during the 3D printing process. Figure 15 illustrates the SEM images of PLA/Mg.

Examiners used X-ray microtomography to analyze microstructures. Examiners tested the tensile mechanical characteristics of all composites and discussed the results in relation to the printing angle conditions. Researchers employed SEM to analyze the fractography of fractured specimens. The study reveals a new way to understand composite materials by measuring how their mechanical properties change based on the printing settings that affect their structure [117]. Researchers collected data using the Taguchi method, specifically L4 (23), and analyzed the data using ANOVA. The nozzle temperature varied between 230 °C and 240 °C, the layer height was between 0.2 mm and 0.3 mm, and the print speed was maintained between 30 mm/s and 40 mm/s. Finding out if the specimens’ hardness changed was the main goal of this study. The findings of the analysis of variance showed that print speed was the most important characteristic, accounting for 56.01% of the total [127]. Examiners found that the printing parameters impacted the hardness of the printed specimens. Examiners achieved a maximum hardness level of 56.3 Shore D by adjusting the print parameters to 240 °C for the nozzle, 0.3 mm for the layer height, and 30 mm/s for the speed. Dentures constructed from PLA–titanium showcased the practical use of this study [116]. The ANOVA results indicate that the layer thickness (*p* = 0.016) and the raster angle (*p* = 0.039) strongly influence the surface roughness of the printed components. This makes selecting the ideal printing parameters to achieve the desired surface roughness effortless. Researchers also assessed the dimensional accuracy of the manufactured component. Researchers looked at thirteen different aspects of the parts, and the FDM machine was very accurate for most of the shapes (with a variation of less than 5%) [128].

The experiments were designed using Taguchi L4 (23), and then S/N ratio evaluation and ANOVA were performed. At an ideal parameter level of 95 °C, a composition of 10/30 g, along with a roller velocity 3.02 mm/s, findings demonstrated that temperature, along with roller speed, remained the most influential parameters on the dimensional accuracy. When it comes to the individual filament tensile examination, the variables that influence it are composition and temperature. With a temperature setting of 100 °C and a roller velocity of 2.70 mm/s, the ideal parameter level is 10/30 g [55]. According to response surface methodology (RSM), the mechanical parameters were affected by a significant parameter. To predict the required output value for different compositions and infill patterns, mechanical qualities studied and mathematically modeled employing the RSM. Finally, the tensile test results show that the octa-spiral pattern is the weakest and the concentric pattern is the strongest for both compositions, with the highest values for elastic modulus and ultimate tensile strength as well as yield strength. A maximum of 0.3 GPa, 8 MPa, and 5 MPa were achieved for the ultimate tensile strength, yield strength, and elastic modulus, respectively. The composite’s tensile behavior was observed to be reduced as the infill composition increased. Another mathematical model that has been developed to facilitate the estimation of tensile characteristics in future FDM printed PLA–brass is an adaptation of RSM [112].

Examiners subjected the specimens to tensile testing and recorded the results in a tabular format. Researchers constructed an ANN model using the NN toolbox in Matlab R2021b (or Scilab) based on these data. Among all the other factors, the most beneficial ones were a high infill density of 40% and a thick-layer thickness of 0.3 mm. The specimen that exhibited the greatest tensile strength was the one with four shells, an infill density of 20%, and a minimum layer thickness of 0.1 mm. Examiners created an ANN model in Matlab using the tensile test results. By comparing the model’s output with experimental data, examiners were able to validate the model’s assumptions about the number of shells, infill density, and random thickness of the layers. Ultimately, the tensile strengths are lower for thicker layers. But the tensile strength goes up with more shells and denser filling. To sum up, examiners were able to effectively construct and evaluate an ANN model for 3D printing aluminum components [129].

### 4.4. Tribological Properties

**Figure 16:**
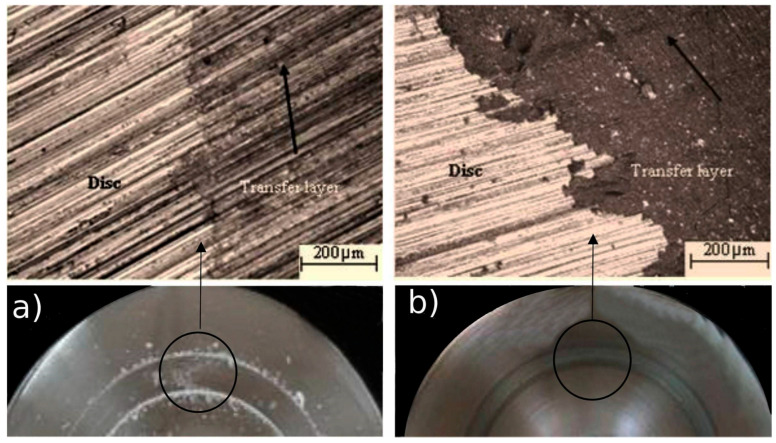
Wear surface morphology of (**a**) pure PTFE, (**b**) PTFE with 60% bronze [120].

A 3D composite filament was fabricated by utilizing the wear properties associated with a PLA/BZ with 14% BZ composite filament being used with the pin-on-disk method. The procedure is carried out by adjusting the load, track diameter, and speed. The gray relational analysis method was employed to maximize the coefficient of friction and minimize wear [130]. The findings showed that the printed end-specimen had a significant impact on the load’s performance. The investigation focuses on load and track diameter, two factors that have a significant impact on mechanical characteristics. The impact of physical characteristics on track diameter become substantial [131]. Figure 16 shows the worn surface morphology of a PLA–bronze biocomposite which depicts the transfer layer and adhesion of the biocomposites to the stainless-steel counterfacing disk.

### 4.5. Biodegradability and Biocompatibility

One way to address the usual constraints of PLA in terms of biodegradation is to incorporate Mg particles into the PLA matrix. It also aids in mitigating alkalinization and excessive H_2_ release, two undesirable outcomes associated with Mg’s quick degradation. Furthermore, the addition of Mg successfully neutralized the acidic by-products produced by PLA, as shown by the change in mass and pH values during In vitro degradation. The biological, microstructural, and thermal characteristics of the scaffolds were evaluated in various studies [132,133]. Biocompatibility depends on the fraction of the metallic particles present in the PLA matrix. The influence of the compounding ratio of SS316L stainless steel on 3D printed PLA biocomposites was evaluated with various percentages and melting temperatures. Maximum mechanical properties were achieved when 12 wt. % of SS316L particles was added, and, at 220 °C, the properties were found to be at their maximum, depicting maximum biocompatibility of the stainless-steel particles [134]. The biocompatibility of the PLA-based biocomposites increased with the addition of Zn nanoparticles for bilayer skin tissue engineering scaffolds by exhibiting high biodegradation rates when exposed to in vivo biodegradation studies, and the biocomposites sustained deep wounds [135,136].

### 4.6. Acoustic Properties

Simulations show that the combined structure with a nickel-coated antenna and a silver-coated diplexer has a return loss of over 10 dB and an excellent gain efficiency of 12 to 17 dBi in the RX band of 10.7 to 12.8 GHz and the TX band of 17.42 to 18.81 GHz, respectively. The measurement findings of the feed system verify these predictions. Additionally, the rejection stage between the TX and RX ports is greater than 60 dB. This low-cost production method for making prototypes or testing ideas for Ku-band systems is shown to be effective because it has a complicated design with many shapes inside, unlike the more expensive CNC-based metal manufacturing methods [122].

### 4.7. Physical Properties

Compared to the untreated samples, ZnOMA-containing compositions showed higher viscosities, which demonstrated surface functionalization-controlled degradation. Researchers found that biocomposites with higher ZnO content had lower viscosity and a more favorable elastic-to-viscous ratio [111]. A new method, which relies on 3D imaging techniques using synchrotron radiation, was used to evaluate the morphology of second-phase iron atoms, including the porosity network inside 3D-printed PLA/magnetic iron composites, at various printing angles. This investigation provided a relationship between these findings and the ductility of the material resulting from fused filament production [137].

## 5. Applications of Pla/Metal Biocomposites

**Figure 17:**
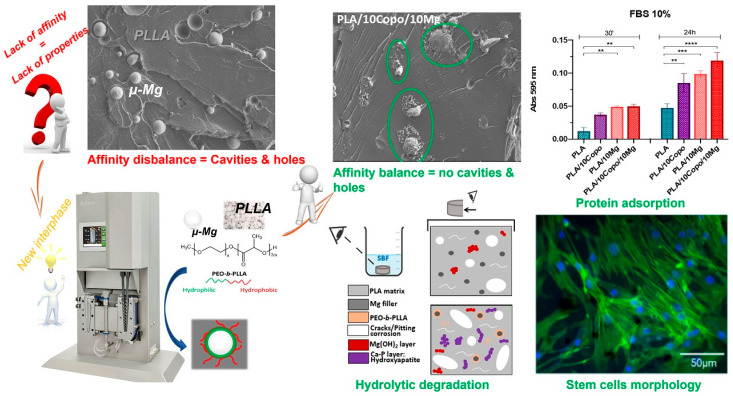
Process of PLA magnesium (** *p* < 0.01; *** *p* < 0.001; **** *p* < 0.0001) [140].

This study demonstrates how interfacial compatibilization plays an essential role in the development of PLA/Mg composites for use in bone regeneration [138]. To facilitate physical connections between the biopolymer matrix and the metallic filler, a synthetic amphiphilic poly-diblock polymer containing a predetermined composition was employed as an interface. (i) Modifying the composite hydrophilicity, bioactivity, and biological behavior and (ii) overcoming the PLA/Mg interface adsorption weakness were both made possible by this strategy. The last step was to conduct stem cell-based biological investigations. The findings demonstrated that the addition of the hydrophilic Mg filler to the hydrophobic PLA matrix increased the interfacial adhesion [139]. The stabilization of the interface was verified by the fact that the damping factor (tanδ) decreased after the copolymer was added. By causing a notable increase in protein adsorption through the specific surface adaptation of the hydrophilic PEO, the latter also demonstrates the advantageous impact of the composite hydrophilicity. In addition, over eight weeks of soaking in the SBF, hydroxyapatite developed in bulk, indicating that its bioactivity is significantly enhanced with the inclusion of the diblock polymer. The ceramic has the potential to act as an inherent binding junction through the osteoregeneration process, connecting the implant to the broken bone. Composite mechanical performances, however, did show a small decline [140]. Figure 17 indicates the entire process of PLA magnesium.

PLA–metal composites widely applied in industry for many applications. PLA–magnesium is used to fabricate anterior cruciate ligament (ACL) screws whereas PLA–zinc (plasma based) is applied in tissue engineering of bone scaffolds at the same time PLA–zinc doped with ZnO and SiC is effectively utilized in triboelectric generators which could transform mechanical energy from their surroundings into electrical energy. PLA–copper is used to fabricate and test dog bone specimens for tensile testing. PLA–bronze is utilized to 3D print items that require a metallic appearance and feel, such as jewelry, statues, artwork, and functional prototypes, and polished to make them shine like metal. PLA–brass is popular for tooling in various applications, like jigs, fixtures, and molds. PLA–titanium is well known for its strength and biocompatibility which could make PLA-based scaffolds and composite filaments for biomedical applications such as bone tissue engineering and orthopedic implants. PLA–aluminum filled filaments are used to print crafts, models. PLA–iron composites find numerous applications, including in magnetic and biological devices. PLA–silver parts could pave the way for the upcoming of low-cost, healthcare, on-demand PPE, and nosocomial devices due to their manufacturing process, their ability to interact with surfaces to prevent adhesion, and their inherent antimicrobial properties. PLA–cobalt is commonly used to create a biodegradable substrate that could work in conjunction with a colorimetric humidity indicator to detect moisture in food packaging [65]. Finally, PLA–nickel composites could create strong and corrosion-resistant parts. These parts could be used in aerospace, automotive, and medical implants. Table 2 shows the various applications of different infill metal with PLA biocomposites.

## 6. Implications

The magnesium particles mixed well with the polymer matrix, and the PLA and magnesium particles did not react chemically. There were no significant differences in the crystallization of the Mg-loaded samples. The cross-sectional pictures of the filaments demonstrate an even distribution of magnesium particles up to a concentration of 15% WE43. Later, the distribution became uneven, pushing the magnesium granules to the edges of the filament cross-section. Additionally, the printing ability of the pores near the magnesium particles changed when the WE43 level increased from 15% to 20%. The low-cost FDM 3D printer demonstrated good printing performance with 5% and 10% WE43 composite filaments [160]. Consequently, these filaments have the potential to create composite biomaterials for bone implant printing. Further studies are needed to assess the biological and mechanical characteristics of these composites. Adding useful functions to ZnO stopped PLA from breaking down and allowed for biocomposites that were more stable at high temperatures to be made. As a bone substitute, 3D-printed porous scaffolds had an elastic modulus that was similar to that of real bone tissue. The amount of ZnO affected all the properties studied, but more time in the plasma did not. The samples that contained 10% ZnOMA had an improved relationship between cellular mineralization and cell longevity, which makes them especially promising for biomedical uses [161].

Looking at the results, examiners saw that above a CuNP concentration of 1%, the virus-killing effect starts to work well. The melting point of the material (Tg) went up as more CuNPs were added, which let us see how the extrusion and additive manufacturing processes affected the results. It became more crystallized after CuNPs were added, which shows that the nanometric particles in this material helped crystallization begin. Thermal analyses with TG and DSC were used to observe this type of activity. In terms of mechanical and thermal properties, the nanocomposites were not as effective as pure PLA [162]. Adding bronze particles to the PLA material strengthened it, which improved its tribological properties. The wear depth significantly decreased compared to previous reports. Even so, the friction stayed the same because the polymer composite matrix was full of rigid particles [75].

In the end, this study looked at the mechanical and physical properties of PLA/brass composites as well as the strength of 3D-printed parts created with FDM. An experiment looked at how different printing settings (layer height, printing speed) and materials affected different mechanical properties. These properties included yield strength, ultimate tensile strength, bending strength, bending modulus, compression strength, compression modulus, and energy absorption. The outcomes indicated that printing parameters and the composition had a big effect on the mechanical characteristics of the PLA/brass composites [163]. Some combinations of printing parameters and specimens made of 15% brass gave the best results for numerous mechanical characteristics. However, examiners found that the optimal mix for impact characteristics was 80 wt. % brass. The layer height and printing speed significantly influenced the Young’s modulus. On the other hand, the composition had the most significant effects on the impact characteristics. These results show how important it is to choose the right printing settings and materials to obtain the best mechanical properties from 3D-printed PLA/brass composites [164]. According to the results, heat treatment could change how the titanium particles are distributed in the PLA matrix, but it does not always make the material stronger when it comes to tensile strength. The dispersed arrangement of particles seen in most specimens indicates that holding at higher temperatures for longer periods of time might cause particles to stick together or additional structural modifications that make the material weaker [165].

The influence of the printing settings on the tensile strength of PLA and PLA/aluminum was evaluated [166]. Three printing settings were changed for the study: the rate of printing (mm/s), the number of shells, and the layer thickness (mm). Taguchi’s DOE was used to print 18 samples, and the tensile test was performed on all of them. The result indicates that PLA has a tensile strength value that is about 20% higher than PLA/aluminum. The reason is that the printed portion of PLA and aluminum was not always the same, which made the bond and structure weak. Both materials’ tensile strength is most affected by the number of shells, according to the ANOVA test. Therefore, examiners determined that the number of shells increases the tensile strength of the FDM-made PLA and PLA/aluminum composite. New recycled iron/PLA composites could be useful in construction, biomedical, and auto companies, where sustainability and low costs are important [167]. This is due to their superior energy absorption and compression capabilities. To improve the mechanical characteristics of these composites even more, future research could investigate how the iron particles are distributed within the polymer and how that affects its compressive strength and power absorption. It could also investigate new material formulations using different fillers, reused iron, and biodegradable polymers. The goal is to create long-lasting 3D-printable composites from recycled and eco-friendly materials that have better properties for a wider range of end-uses [168]. It was stated in some Life-Cycle Analysis (LCA) studies that the PLA-based composites end up in composting or landfills after their End-of-Life (EoL). The greenhouse gas emissions in each stage of the utilization of PLA were analyzed, including the acquisition and conversion of the feedstocks, manufacturing of PLA biocomposites, their applications, and finally EoL conditions. Conversion from the feedstock was evaluated to be most energy intensive process in the lifecycle of PLA. If the conversion process was optimized to consume low energy, then PLA could be a potential low carbon material even at its EoL [169,170].

Some other studies have stated that metal ingredients and polymeric compounds could kill *E. coli* and *Listeria monocytogenes* bacteria during all stages of industrial production. At a concentration of 2%, the additive R148 demonstrates its maximum effectiveness in killing microbes on surfaces, indicating its potential for use in medicine [171]. This result suggests that the CoCl-coated 3D substrate could be useful for monitoring the humidity of products. This technology also offers benefits when applied to other polymers, fostering innovative and environmentally friendly ideas. For instance, examiners can use it continuously at high frequencies and in small amounts. It also allows for very precise ink drop patterning [65]. According to the feed system measurements, the combined design of the nickel-coated antenna and silver-coated diplexer shows an extra 10 dB of reverse damage and an improvement of 12 to 17 dB in the RX band (10.7 to 12.75 GHz) and the TX band (17.4 to 18.8 GHz). The isolation range among the TX and RX ports is also more than 60 dB. While CNC-based metal production is pricier, this low-cost method might be utilized for prototype constructions or proof-of-concept studies of Ku-band schemes, as shown by the complex structure with many detailed shapes inside.

### 6.1. Limitations

The poor dimensional accuracy and large tolerances of manufactured items from inexpensive 3D printers pose a significant problem [172]. Improving the exterior roughness of FDM-made components necessitates exploring various printing parameters, including printing speed and layer depth, as well as the scanning angle. Therefore, the goal of this study is to examine the effectiveness of various FDM surface finish parameters, including printing speed and layer thickness, as well as the raster angle. The experimental design was based on Taguchi’s approach, which uses the L9 array to reveal the response. Researchers created the prototype model according to ISO guidelines using PLA–aluminum filament [128].

The short processing window and inferior mechanical characteristics of PLA make it less practical. PLA was used to develop composites to enhance its qualities for FFF printing [173]. To successfully manufacture composite components using this technology, it is essential to establish a relationship between the process parameters and the pieces’ mechanical qualities. Composite parts sectors like automotive, aerospace, and medical rely on high-performance composite materials. To optimize the printing parameters to obtain an appropriate mechanical qualities is critical. Controlling and forecasting the mechanical properties of FFF-printed composite parts is crucial for their successful integration into these industries.

### 6.2. Future Prospects

To better understand the potential for future research on FDM, researchers conducted a critical literature review to gather information about the manufacturing and maintenance of this hybrid material employing different methods and methodologies. The main goal of this review is to learn about the advancements in creating different PLA/Mg composite mixtures for making implants, with the aim of producing cost-effective and flexible prosthetics using 3D printing. This review looks at articles published in English from 2016 to 2020 that talk about fused deposition modeling and magnesium-polylactide composites to link the use of PLA/Mg as a filament or base material with the growing medical applications of fused deposition modeling [174].

As a result, plasma functionalization was able to circumvent ZnO’s detrimental thermal degradation impact on PLA, paving the way for the additive manufacturing of bioactive scaffolds that hold immense promise for use in tissue engineering projects [162]. A novel fibrous composite material was developed, and its antimicrobial and physicochemical characteristics were studied [175]. The process involves creating the nonwoven fabric and then treating it in two steps: first, soaking the samples in a zinc (II) chloride solution, and second, soaking them in a mixture of alginic sodium salt. Specific surface area, total/average pore volume, and SEM were all used in the characterization and analysis of the new material. To check how well the polylactide/alginate/Zn fibrous composite could fight germs, it was tested on different bacteria, like *Staphylococcus aureus*, *Escherichia coli*, and two kinds of fungi [176]. These findings provide the groundwork for future research into the creation and possible use of a novel composite material with antimicrobial and antifungal properties in the biomedical field [177]. The present investigation aims to close this information gap by assessing how bronze content and 3D printing direction affect the tribological characteristics of the bronze/PLA composite [75].

#### Envisage Using the Patent Landscape for PLA Metal Composite

**Figure 18:**
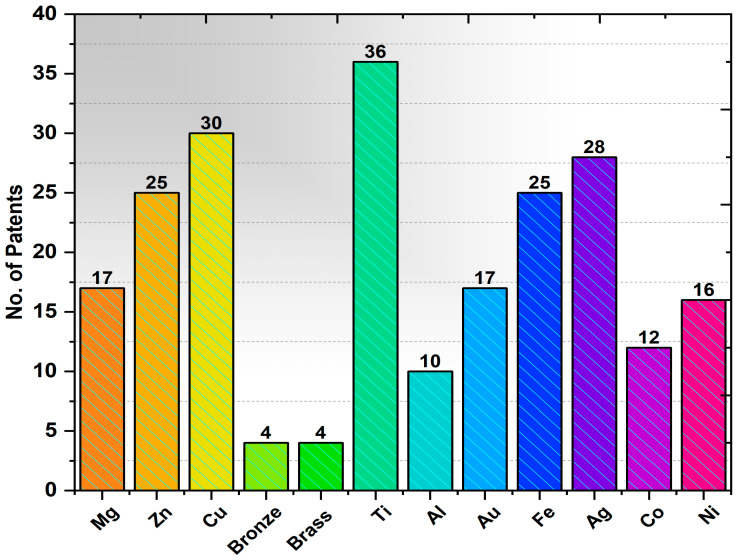
Number of patents filed with PLA combined with Metal.

Patent landscape analysis provides a comprehensive understanding of future growth. While searching, the following options are used: it was searched only on “English claim”, single family member, and stemming; the count of patents filed is shown in Figure 18. The keywords used were PLA with individual metal analyzed along with “3D printing”. Figure 18 reveals that the PLA with metal or the alloy of the specific metal composite has favorable growth owing to its contributing to many applications. The researchers can focus on this PLA–metal combination for versatile new uses because only the patent claims are analyzed to ensure inventiveness. Figure 18 indicates the number of patents filed with PLA in combination with metal.

It was also understood that various patents for different applications were filed in PLA and metal combinations. PLA-based bimetallic polymer materials were developed using copper and aluminum particles and were employed for safe handling and enhanced antimicrobial properties. PLA was combined with synthetic glass powders rich in zinc and magnesium and biofunctionalized composites were developed for scaffold and biofunctional applications.

## 7. Summary and Conclusions

The addition of metallic particles as fillers to PLA-based biocomposites is an ongoing research trend owing to their numerous advantages, including enhanced properties and wider application spectrum of the composites. The purpose of this research was to see whether adding silver particles to PLA changed the surface properties of the printed components compared to using regular PLA. Researchers subjected 3D-printed specimens of commercial PLA with silver particles added for antibacterial qualities to experiments that included SEM and image analysis, FTIR, and surface wetting. The data revealed no discernible alterations in surface morphology or chemistry, despite the addition of silver particles causing an uneven layer thickness in the part’s structure [178]. This review article gives the in-depth analysis of PLA with metal nanoparticles printed by fused deposition modeling. Filament extrusion for various PLA–metal-printed specimens was discussed along with printing parameters. Several properties like tensile, flexural, compression, Young’s modulus, impact, hardness, ductility, ultimate tensile strength, and wear are discussed here.

Various optimizations like ANOVA, Grey analysis, X-ray microscopy, scanning electron microscope, thermal stability, and biodegradation of PLA Metal composites are also discussed here. Three-dimensional printing with PLA–metal composite filaments is not always simple because the two materials have different characteristics. Filament fragility, increased brittleness, and an uneven distribution of metal particles could cause problems with nozzle clogging and breakage. Metal particles are abrasive, which wears down nozzles and stresses extruder components. Additionally, controlling flow and temperature becomes more complicated due to different thermal properties. The print speed is affected by the extra weight, and warping could occur due to differences in cooling rates. The material is expensive and scarce, and post-processing necessitates extra attention to surface quality. Specialized hardware, optimized print settings, and a supervised printing facility might help mitigate these challenges. The patent landscape reveals the significant growth for this composite material with the potential ability for manufacturing.

## Figures and Tables

**Table 1:** Three-dimensional printing process parameters for manufacturing PLA/Metal biocomposites.

S.No.	Biocomposite	AM Technique	Process Parameters	Reference
1	PLA with 5–20% Mg biocomposites	FDM	Nozzle diameter—0.4 mm, Layer thickness—0.2 mm	[46]
2	PLA coated AM60 alloy biocomposites	FDM	Nozzle temperature—245 °C and bed temperature—60 °C, Infill speed—60 mm/s, Nozzle diameter—0.4 mm, Layer thickness—50 µm	[83]
3	PLA with 15% and 80% of brass biocomposites	FDM	Layer height—0.25—0.35 mm, Printing speed—20–40 mm/s, Infill percentage—50%	[84]
4	PLA Copper biocomposites	FDM	Nozzle temperature—200–230 °C, Bed temperature—70–110 °C, Printing speed—40 mm/s	[85]
5	PLA with Copper biocomposites	FDM	Nozzle diameter—0.4 mm, Layer height—0.2 mm, Nozzle temperature—215 °C, Bed temperature—60 °C	[86]
6	PLA with Silver biocomposites	FDM	Nozzle diameter—0.4 mm, Nozzle temperature—220 °C, Printing speed—90 mm/s	[87]
7	PLAwith AgNPs biocomposites	FDM	Nozzle diameter—0.4 mm, Layer height—0.2 mm, Printing speed—90 mm/s, Infill percentage—75%, Nozzle temperature—220 °C,	[88]
8	PLA with Iron microparticles biocomposites	FDM	Gyroid infill pattern, Nozzle temperature—215 °C, Bed temperature—60 °C, Printing speed—70 mm/s,	[89]
9	PLA with 10% of 316 L stainless-steel biocomposites	FDM	Filament diameter—2.85 mm, Screw speed—30 rpm, Nozzle temperature—185 °C	[90]
10	PLA with Cobalt ferrite nanoparticle biocomposites	FDM	Nozzle temperature—260 °C, Bed temperature—60 °C, Printing speed—20 mm/s	[91]
11	PLA with Nickel biocomposites	FDM	Layer thickness—0.15 mm, Print speed—10 mm/s, Infill—100%	[92]

**Table 2:** Applications of different metal infill in PLA biocomposites.

Sl.No	Metal Infill	Applications	Ref.
1.	Mg	Bone Tissue Engineering Scaffolds	[141]
2.	Mg	3D-Printable PLA/Mg Composite Filaments	[46]
3.	Zn	Antibacterial Orthopedic Implants	[142]
4.	Zn	Direct Ink Writing (DIW) for Scaffold Fabrication	[143]
5.	Zn	Surgical Tools, Orthopedic Implants, Dental Applications	[144]
6.	Cu	Food Packaging Materials	[145]
7.	Cu	LED Housings	[146]
8.	Bronze	Electromagnetic and Magnetic Applications,	[147]
9.	Bronze	Fit and Form Testing	[148]
10.	Bronze	Art, Sculpture, and Jewelry	[149]
11.	Brass	Used in Investment Casting Workflows	[150]
12.	Ti	Custom Gadgets, Phone Cases, Eyewear Frames	[151]
13.	Fe	Sensing, and Magnetic Devices.	[152]
14.	Fe	Biomedical Engineering	[153]
15.	Ag	Low-Cost Antimicrobial Surgery Equipment	[154]
16.	Co	Supercapacitors	[155]
17.	Co	Surface Modification and Functional Coatings	[156]
18.	Ni	Porous Structures	[157]
19.	Ni	Catalysis	[158]
20.	Ni	Electromagnetic Wave Absorption	[159]

## Data Availability

No new data generated or created.
